# The Impact of Fermentation on the Antioxidant Activity of Food Products

**DOI:** 10.3390/molecules29163941

**Published:** 2024-08-21

**Authors:** Sümeyye Sarıtaş, Alicia C. Mondragon Portocarrero, Jose M. Miranda López, Mauro Lombardo, Wojciech Koch, António Raposo, Hesham R. El-Seedi, José Luiz de Brito Alves, Tuba Esatbeyoglu, Sercan Karav, Anna Maria Witkowska

**Affiliations:** 1 Department of Molecular Biology and Genetics, Çanakkale Onsekiz Mart University, Çanakkale 17000, Türkiye; sumeyyesaritas@stu.comu.edu.tr; 2 Laboratorio de Higiene Inspección y Control de Alimentos, Departamento de Química Analítica, Nutrición Bromatología, Universidade de Santiago de Compostela, Campus Terra, 27002 Lugo, Spain; aliciamondragon@yahoo.com (A.C.M.P.); josemanuel.miranda@usc.es (J.M.M.L.); 3 Department for the Promotion of Human Science and Quality of Life, San Raffaele Open University, Via di 11 Val Cannuta 247, 00166 Rome, Italy; mauro.lombardo@uniroma5.it; 4 Department of Food and Nutrition, Medical University of Lublin, 4a Chodźki Str., 20-093 Lublin, Poland; kochw@interia.pl; 5 CBIOS (Research Center for Biosciences and Health Technologies), Universidade Lusófona de Humanidades e Tecnologias, Campo Grande 376, 1749-024 Lisboa, Portugal; antonio.raposo@ulusofona.pt; 6 Chemistry Department, Faculty of Science, Islamic University of Madinah, P.O. Box 170, Madinah 42351, Saudi Arabia; elseedi_99@yahoo.com; 7 Department of Nutrition, Health Science Center, Federal University of Paraíba, João Pessoa, PB 58051-900, Brazil; jose.luiz@academico.ufpb.br; 8 Department of Molecular Food Chemistry and Food Development, Institute of Food and One Health, Gottfired Wilhelm Leibniz University Hannover, Am Kleinen Felde 30, 30167 Hannover, Germany; esatbeyoglu@foh.uni-hannover.de; 9 Department of Food Biotechnology, Bialystok Medical University, 15-089 Bialystok, Poland

**Keywords:** antioxidant activity, fermentation, fermented food, functional food, dairy products

## Abstract

From ancient times to the present day, fermentation has been utilized not only for food preservation but also for enhancing the nutritional and functional properties of foods. This process is influenced by numerous factors, including the type of microorganisms used, substrate composition, pH, time, and temperature, all of which can significantly alter the characteristics of the final product. Depending on the parameters, fermentation enhances the bioactive content of the products and imparts the necessary properties, such as antioxidant characteristics, for the products to be considered functional. The enhancement of these properties, particularly antioxidant activity, enriches foods with bioactive compounds and functional attributes, contributing to improved health benefits. Through a review of recent research, this study elucidates how different fermentation processes can enhance the bioavailability and efficacy of antioxidants, thereby improving the nutritional and functional qualities of foods. This study investigated the multifaceted effects of fermentation on antioxidant properties by exploring various types and conditions of fermentation. It highlights specific examples from dairy products and other food categories as well as the valorization of food waste and byproducts. The findings underscore the potential of fermentation as a sustainable method to produce health-promoting foods with elevated antioxidant activities, offering new perspectives for food science and technology.

## 1. Introduction

Fermentation, a technique used for centuries to preserve food, has recently attracted increased amounts of attention due to the increasing interest in healthy nutrition and its many advantages. This metabolic process is invaluable because of its low energy cost and ability to enhance and preserve the properties of the product [1]. It is known that, through this process, the nutritional value of food products can be enhanced along with improvements in their sensory properties and the incorporation of functional properties into food products [2,3]. Fermentation represents a safe process that can improve the sensory properties, nutritional value, and acceptability of food products. Fermented food products may exhibit various features, including antimicrobial, anti-inflammatory, and antioxidant activities, due to the release and generation of phenolic compounds, flavonoids, and peptides, as well as increased digestibility [4,5]. Fermentation improves the bioaccessibility, bioavailability, biofunctionality, and bioactivity of products [6,7]. Additionally, it can enhance the metabolic profile of food products [8].

Fermentation has a significant impact on the antioxidant activity of food products. Through this process, bioavailability, which refers to the ability of a compound to demonstrate its biological functions after being absorbed by the body and entering the circulation, is enhanced [9,10]. High bioavailability is associated with increased antioxidant activity. Bioavailability enhancement is mainly attributed to the degradation and release of bioactive compounds through the fermentation process [11]. Fermentation can enhance antioxidant function in three main ways: releasing, promoting, and producing antioxidant compounds [12,13].

Milk and dairy products are known for their benefits, consumed from childhood to old age thanks to their rich content [14]. These products attract attention with their rich protein, fat, and carbohydrate content. They are preferred for functional food production due to both the benefits provided by fermentation and the benefits provided by their content [15,16,17]. As a result of fermentation, the products are included in the functional food group with health-promoting effects, including antioxidant activity [18]. They have antioxidant properties, including mainly enhanced phenolic contents, and exhibit scavenging activities [5,19].

Antioxidants have been utilized for centuries to protect human health against damage caused by harmful molecules known as free radicals [20,21]. These detrimental molecules contribute to the development of various diseases, including metabolic diseases, heart disease, and cancer [22,23]. Therefore, the consumption of foods rich in antioxidants plays a crucial role in maintaining and improving human health [24]. The general functions of antioxidants can be summarized as follows: (1) protecting against oxidative stress, (2) supporting the immune system, (3) reducing the risk of chronic disease, (4) protecting against aging, and (5) promoting overall health. Additionally, fermentation can be utilized to produce various types of food products [4,21,25]. The consumption of fermented food products promotes human health due to their antioxidant activity, which is based on phenolic compounds and flavonoids [26,27].

This review explored the relationship between fermentation and antioxidant activity based on related research. This work investigated the effects of fermentation type and conditions on antioxidant properties and examined the impact of fermentation on the production of antioxidant-rich foods. The focus was on dairy products and various other foods, comprehensively addressing how fermentation enhances their nutritional and functional qualities in terms of antioxidant properties.

## 2. Understanding Fermentation

Bioconversion is the process of transforming a compound from one form to another using microorganisms [28]. Fermentation is a useful method for bioconversion because it can convert carbohydrates, such as sugars and starches, into alcohol and organic acids [29,30]. The process of fermentation begins with the breakdown of carbohydrates and organic compounds by microorganisms, such as yeast, lactic acid bacteria (LAB), or other beneficial bacteria [29]. These microorganisms aid in the fermentation of carbohydrates in the product, resulting in the production of organic acids, alcohol, and carbon dioxide [9,31].

**Figure 1:**
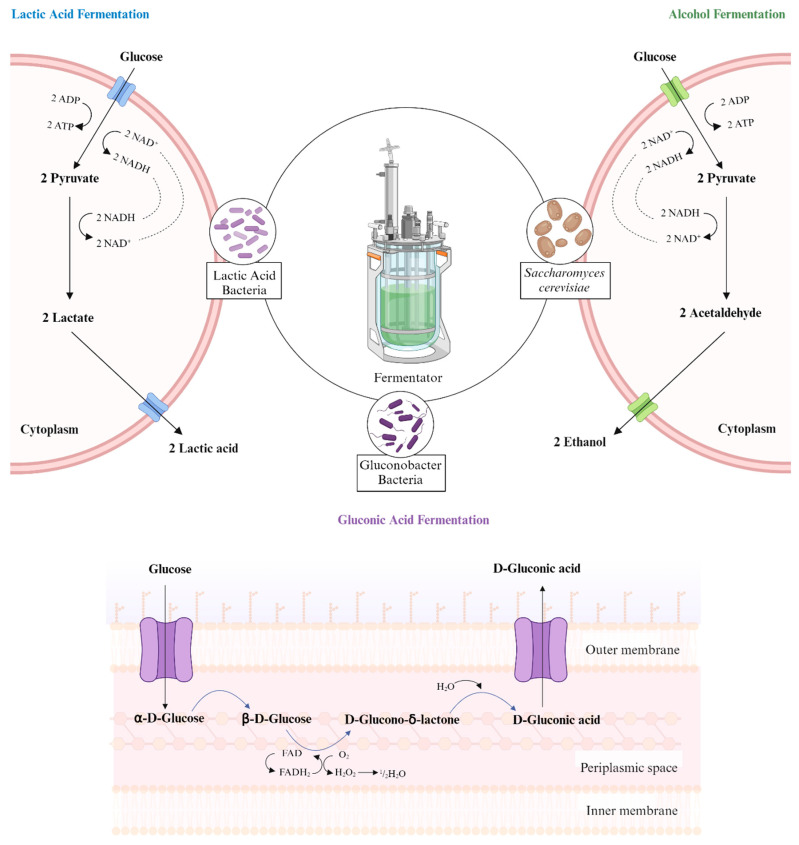
Illustration of three types of fermentation [15,35].

There are various types of fermentation, including alcohol, LAB, solid-state, and liquid fermentation (Figure 1) [32,33]. The two most well-known types are LAB fermentation and alcohol fermentation, which require specific microorganisms [15]. It is important to choose the appropriate type of fermentation, as it directly affects the end product and application areas [15]. After obtaining the end products, which are based on the type of fermentation, they can be used in various processes, including flavor and odor creation or other metabolic processes [34].

The transformation of glucose to pyruvate, known as glycolysis, involves multiple steps [36]. In the cytoplasm, glycolysis occurs, resulting in the production of pyruvate molecules and cellular energy, while NAD^+^ molecules are reduced to NADH molecules [36]. The fundamental steps of fermentation are similar across the types of fermentation [15].

Alcohol fermentation is a process that involves yeast and bacteria converting carbohydrates into end products, including ethanol (EtOH), acetic acid, and carbon dioxide [37]. In the absence of oxygen, pyruvate molecules are transformed into acetaldehyde and carbon dioxide [37]. Acetaldehyde is then converted into ethanol, with NADH molecules being oxidized to NAD^+^ molecules in the process. The process of alcohol fermentation concludes with the production of the end products [37]. Alcohol fermentation typically occurs with yeast species, primarily *Saccharomyces cerevisiae* (*S. cerevisiae*) [38]. *S. cerevisiae* is commonly used in beverage production because of its high alcohol tolerance and adaptability to various fermentation conditions [38]. A study conducted by Czabaj et al. demonstrated that the sugar content of mead beverages is altered after alcohol fermentation, indicating that the microorganisms involved in fermentation utilize sugar [39,40].

LAB are commonly employed in fermentation to enhance the nutritional, functional, and sensory properties of food products [20,41]. The fermented product provides benefits to human health due to the functions of LAB strains, including probiotic properties [42,43]. LAB fermentation, a popular type of fermentation, involves certain bacteria known as LAB. Fermentation occurs in the cytoplasm and begins with the transformation of glucose to pyruvate [36]. In this step, NAD^+^ molecules are reduced to NADH molecules, and cellular energy is produced [36]. Then, the pyruvate molecules are converted into lactate, promoting the formation of lactic acid [36]. During the production of lactic acid, NADH molecules are oxidized to NAD^+^ molecules [36].

Studies have demonstrated that different bioactive molecules can be enhanced by LAB, which have various characteristics [31,44]. In a study, which was conducted by Duan et al. (2023), changes in the sensory quality, including the aromatic profile, taste, odor, and color, of goji juice after LAB fermentation were examined. The results revealed an increase in the sensory quality of the LAB-fermented product [31]. In a study conducted by Wu et al., apple juice was fermented by LAB strains. Malolactic bioconversion, which involves the conversion of malic acid to lactic acid by bacterial strains, occurs during fermentation. LAB strains consumed malic acid during fermentation and produced lactic acid. As a result of malolactic bioconversion, changes in the pH of apple juice were observed [45]. Similarly, when different African nightshade leaves were fermented by LAB, pH changes occurred during fermentation [42].

Physicochemical properties, metabolomic profiles, and antioxidant properties of wolfberry longan juice after fermentation with *Lacticaseibacillus paracasei* and *Lactococcus lactis* subsp. *lactis* [46] were noticed. Similar to other studies, LAB fermentation reduced the sugar content and affected the color of the juice. The phenolic and flavonoid contents increased after fermentation. LAB fermentation also caused alterations in the metabolite profile, with 193 metabolites upregulated and 181 metabolites downregulated by LAB fermentation [46].

LAB can metabolize materials present in products and break down and utilize materials specific to their different species and strains [46]. The metabolic capacity varies among species due to their distinct metabolic pathways and enzymes. The metabolism of LAB is influenced by the availability of fermentable nutrients and phenolic compounds in the environment [47]. Phenolic compounds can exert both beneficial and negative effects on LAB metabolism. In particular, high levels of phenols may inhibit LAB activity, affecting their metabolism and viability [20]. Therefore, the selection of LAB strains that can tolerate high phenolic levels is crucial. During fermentation, LAB secrete various enzymes including β-galactosidase, proteases, and peptidases to degrade and hydrolyze the components of the fermented product, thereby enhancing productivity through the release of these compounds [20]. Additionally, in a study conducted by Martí-Quijal et al., *Lactobacillus plantarum* (*L. plantarum*) isolated from sea bass demonstrated significant proteolytic capacity and the ability to synthesize phenolic compounds that affect antioxidant activity [48]. Li et al. revealed that, after fermenting edible grass with *L. plantarum* and *Lactobacillus rhamnosus* (*L. rhamnosus*), the antioxidant activity, soluble protein content, and organic acid content of edible grass increased [49].

Solid-state fermentation is a type of fermentation in which microorganisms metabolize and grow using solid substrates [50,51,52]. In a study by Xiao et al., dark tea was fermented using *Bacillus subtilis* (*B. subtilis*) [53], and it was found that this bacterial species plays an essential role in metabolizing volatile and nonvolatile organic compounds in dark tea [53]. Aroma and flavor, two critical properties of foods, were also found to be improved by solid-state fermentation [53]. Similarly, after the fermentation of corn bran, the phenolic content is improved according to studies by Akbari et al. [54]. Interestingly, there was a slight decrease in polyphenol content after fermentation, although antioxidant properties were still retained [53]. In another study, after the solid-state fermentation of dandelion, its flavonoid content, composition, and antioxidant activity were investigated [1]. The 2,2-diphenyl-1-picrylhydrazyl (DPPH) radical scavenging activity and reducing power measurements were conducted in vitro to compare fermented and unfermented dandelion extracts. The fermented samples exhibited increased antioxidant activity [1]. These findings suggest that solid-state fermentation enhances the bioavailability of dandelion [1].

In another study in which apple pomace was fermented with the white rot fungus *Phanerochaete chrysosporium* via solid-state fermentation, changes in the antioxidant content were examined [11]. The effectiveness of three different fermentation methods—flasks, trays, and bioreactors—was compared [11], and additionally, ethanol and acetone were used to determine how the choice of solvent affected the extraction of polyphenolic compounds [11]. The results showed that the free radical inhibition capacity of polyphenolic compounds reached its highest level on the seventh day. A higher polyphenol content was obtained when acetone was used as the solvent. The polyphenol content in the tray and bioreactor methods was significantly greater than that in the flask fermentation method. The free radical inhibition capacity of DPPH increased more significantly when acetone was used as the solvent. Furthermore, this study aimed to monitor enzyme activity during the fermentation period, focusing on β-glucosidase, an enzyme that facilitates the release of polyphenolic compounds. During solid-state fermentation, β-glucosidase activity increased significantly between the sixth and eighth days. β-glucosidase activity was greater in the tray and bioreactor methods than in the flask method. Additionally, the production of lignocellulose-degrading enzymes gradually increased throughout the fermentation period. Taken together, the results of this study demonstrated that apple pomace, which is typically considered a waste byproduct, can be transformed into a valuable product with enhanced polyphenol content and increased antioxidant properties through solid-state fermentation. This innovative approach not only improves the nutraceutical value of apple pomace but also contributes to environmental sustainability [11].

A study conducted in 2020 aimed to evaluate the nutritional composition, protein quality, antinutritional component activity, and antioxidant activity of soy flour after solid-state fermentation [55]. For this purpose, changes in the protein, fat, and fiber content of soy flour fermented with *L. casei* were examined. The Kjeldahl method was employed to determine the protein content, calculating the amount of protein by assessing the nitrogen content in the sample. The results revealed an increase in protein content following fermentation. However, there was a significant decrease in the crude fiber content after fermentation [55]. Total and free amino acid contents were greater before fermentation than after fermentation. Additionally, DPPH and hydroxyl radical scavenging activities, as well as the ferrous ion chelating ability, increased rapidly within the first 48 h of fermentation.

Another kind of solid-state fermentation is pile fermentation, commonly known in compost production [56]. The term “pile fermentation” refers to the use of piled-up materials to enhance the fermentation efficiency of organic compounds [57]. Similar to LAB fermentation, pile fermentation occurs through the same pathways and by LAB. However, in pile fermentation, different types of microorganisms can be used in addition to LAB. In a study conducted by Zhang et al., aiming to evaluate the antioxidant activity of Pu-erh tea resulting from pile fermentation, the antioxidant capacities of tea extracts at various degrees of pile fermentation were determined by measuring the DPPH radical and hydroxyl radical scavenging activities [57]. The study revealed that the antioxidant properties of Pu-erh tea water extracts are influenced by the degree of fermentation, which affects both DPPH radical and hydroxyl radical scavenging activities [57].

Gluconic acid fermentation occurs in the periplasmic space and involves the fermentation of glucose or glucose-containing substances by bacteria of the *Gluconobacter* genus to produce gluconic acid [35]. These bacteria typically operate under alkaline conditions and carry out oxidative fermentation [35]. In a study by Hornedo-Ortega et al., the effects of alcohol and gluconic fermentation types on the preservation of anthocyanin compounds, a type of antioxidant, in strawberry beverages were compared [3]. Gluconic fermentation provided better preservation of anthocyanin compounds than alcoholic fermentation [3]. After gluconic fermentation, it was observed that the beverages increased cell viability and even reduced oxidative stress induced by amyloid-β peptide [3].

Throughout history, fermentation has been used to preserve many foods [58]. Fermentation, which involves various processes that transform substrates into valuable end products by the action of microorganisms, has been used to extend the shelf life of foods, increase their nutritional value, and increase their functional properties [12,59]. Various types of fermentation, such as alcohol, LAB, and gluconic fermentation, provide benefits in food and beverage production because the end products are produced depending on the type of fermentation [60,61]. 

## 3. Impact of Fermentation on Antioxidant Activity

Fermentation provides a range of benefits, including the preservation of foods, especially dairy products, the enhancement of bioactive content, and the improvement of nutritional functions [62,63]. Fermentation helps improve the antioxidant properties of foods through five main pathways. The first is enhancing the phenolic content of foods; the second is increasing the production of metabolic products; the third is enhancing the synthesis of antioxidant compounds; the fourth is enhancing enzymatic activity; and the last is converting phytochemical compounds into more active antioxidant forms [24,64,65]. In particular, bioactive peptides can be obtained through proteolytic activity or enzymatic hydrolysis by fermentation [66,67]. Antioxidative activity includes radical scavenging effects, such as hydrogen donation and free radical scavenging properties, metal ion chelation, and inhibition of lipid peroxidation [16,67].

During fermentation, a variety of enzymes are responsible for producing and promoting the release and synthesis of bioactive compounds from the substrate [1]. The increase in polyphenol, phenolic, and flavonoid contents released during fermentation enhances antioxidant activity [2,68,69]. Fermentation can enhance the antioxidant properties of red cabbage by releasing phenolic compounds bound to the cell walls. This finding provides evidence that the release of bioactive components through fermentation can increase antioxidant properties [29]. Similarly, the total phenolic content of strawberry juice fermented with *L. plantarum*, and *Lactobacillus delbrueckii* subsp. *acidophilus* (*L. delbrueckii* subsp. *acidophilus*) increased compared to that of unfermented strawberry juice. Additionally, the fermented product exhibited greater antioxidant properties [70]. However, fermentation led to a decrease in the total phenolic and flavonoid content [28,49,70].

In a work reported by Zhou et al., the fermentation of kiwifruit pulp with *L. plantarum* was investigated, and the effects of fermentation on the phenolic profile, antioxidant activities, and metabolites of the fruit were examined. The increase in phenolics and flavonoids after fermentation was associated with a radical scavenging effect. Comparisons between nonfermented and fermented samples revealed metabolic differences in some components of the samples. These components included lactic acid, fructose, phosphoric acid, gluconolactone, and the sugar of kiwifruit pulp [2].

On the other hand, fermentation can improve radical scavenging activity through antioxidant activity [71,72]. In a study conducted by Pontonio et al., 20 varieties of pomegranate were used to evaluate the variety of products and how the fermentation process influences antioxidant activity, bioavailability, and bioaccessibility [73]. Fermentation can influence the phenolic content and radical scavenging activities of pomegranate juice fermented by *L. plantarum* [73]. Similarly, after fermentation, a *Porphyra yezoensis* (*P. yezoensis*) sauce demonstrated DPPH scavenging activity and ferric reducing ability (FRAP) [74].

Beyond the question of how to improve the antioxidant activity of products via fermentation, the effects of fermentation on antioxidant activity are influenced by various parameters.

### 3.1. Factors Influencing the Impact of Fermentation on Antioxidant Activity

**Figure 2:**
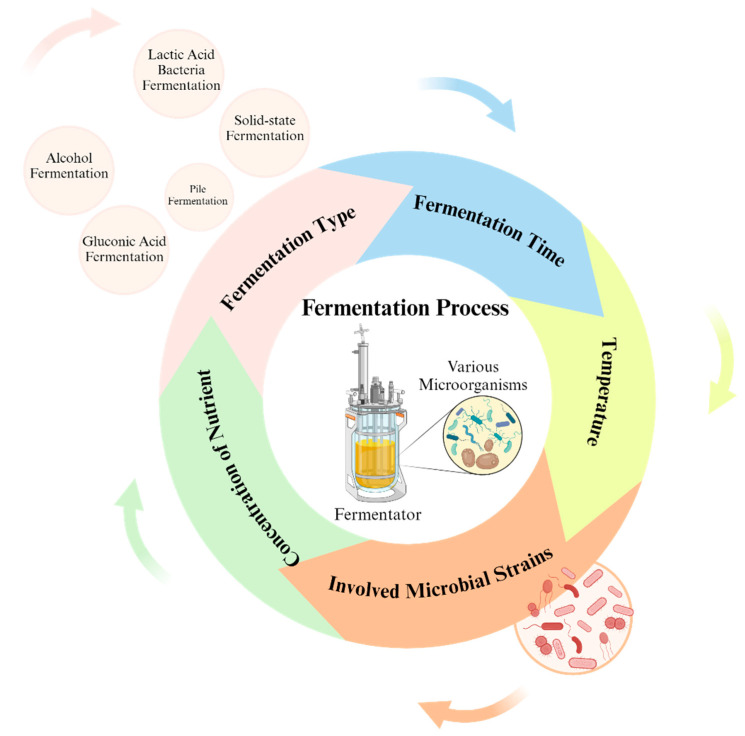
Factors affecting the impact of fermentation on the antioxidant profile.

Several factors influence the impact of fermentation on antioxidant activity, including fermentation type and microbial strains involved in fermentation (Figure 2) [75].

#### 3.1.1. Microbial Strain

The fermentation process is influenced by the microorganisms involved. In studies using different LAB strains, it was observed that phenolic compounds changed after fermentation depending on the strain [76,77]. Hunaefi et al. investigated the variation in the antioxidant properties of red cabbage fermented with *L. plantarum* and *L. delbrueckii* subsp. *acidophilus* [29]. Compared with the unfermented samples, the fermented samples exhibited greater antioxidant activity. Additionally, red cabbages fermented with *L. plantarum* exhibited greater antioxidant activity. This suggests that various bacterial species within the same genus may not have equal impacts on product properties following fermentation.

A recent study conducted by Kozłowska et al. aimed to increase the nutritional content of fermented milk by incorporating young barley leaf powder [78]. For this purpose, young barley leaf powder was introduced into milk fermentation processes employing various starter cultures. This study investigated the impact of both young barley powder and starter cultures containing different LAB on the fermentation process and subsequent storage of the product. The acidification patterns observed during LAB fermentation were found to be distinct, contingent upon the specific starter culture utilized. Additionally, it was noted that acidity levels during the storage of fermented dairy products varied according to the type of starter culture employed. Furthermore, this study revealed that the inclusion of young barley leaf powder in milk fermentation had a positive influence on bacterial growth rates and enhanced the quality parameters of the resulting fermented milk product. Taha et al. aimed to evaluate the antioxidant and antibacterial activities of yogurts fermented with different starter cultures using buffalo milk [47]. For this purpose, in this study, which was conducted with three different groups of samples, the first group served as the control group and was fermented using yogurt culture (*Streptococcus salivarius* subsp. *thermophilus* and *Lactobacillus delbrueckii* subsp. *bulgaricus*). On the other hand, the second group of samples was fermented using *L. delbrueckii* subsp. *acidophilus* in addition to yogurt culture. The third group was fermented using *Lactobacillus helveticus* in addition to yogurt culture. Lactic acid fermentation significantly increased the antioxidant activity of the yogurt samples during fermentation and cold storage. According to the 2,2′-azino-bis(3-ethylbenzothiazoline-6-sulphonic acid) (ABTS) test results, the highest level of antioxidant activity was observed in the control group, followed by the second group and then the third group. According to the DPPH test results, the third group exhibited the highest antioxidant activity, followed by the second group and the control group. Both test results indicated that the antioxidant properties increased for all the samples during cold storage.

#### 3.1.2. Fermentation Time

The time of the fermentation process affects the properties of the product after fermentation, especially the vitality of the microorganisms that provide fermentation. Fermentation time significantly impacts antioxidant activity due to the slow adaptation and growth of the fermentation strain used during the initial stages [79]. As fermentation proceeds, metabolic activities and the synthesis of bioactive compounds increase as the strains grow [80]. Insufficient fermentation time may lead to lower antioxidant activity, whereas excessively prolonged fermentation may result in a series of problems, including nutrient depletion, that can negatively affect antioxidant activity [80]. In the study conducted by Lasinskas et al., the changes in the antioxidant properties of fireweed (*Chamerion angustifolium*) leaves before and after fermentation were investigated with a focus on the effect of fermentation time. Fireweed leaves fermented for 24, 48, and 72 h exhibited the greatest increase in antioxidant activity after 72 h of fermentation [81]. In the study by Jakubczyk et al., the fermentation of kombucha was carried out by symbiotic cultures of bacteria and yeasts. The study revealed that the fermentation time, type of microorganism, and type of tea used affected the antiradical and antioxidant properties [82]. Another study aimed to purify and characterize novel antioxidative peptides derived from goat milk fermented by *Lactobacillus fermentum* (*L. fermentum*) [83]. Researchers have evaluated antioxidant activity in fermented goat milk and optimized growth conditions, yielding the highest activity, and subsequently purified and characterized the peptides produced. Through experiments conducted at various incubation times (0, 12, 24, 36, and 48 h), it was determined that the highest antioxidant activity was observed after 48 h of fermentation. These findings were validated through the ABTS assay, hydroxyl free radical scavenging assay, and superoxide free radical scavenging assay. Protein and peptide analysis was performed using SDS–PAGE and two-dimensional gel electrophoresis (2D gel electrophoresis), followed by peptide separation and identification using reverse-phase high-performance liquid chromatography (RP–HPLC) and liquid chromatography–mass spectrometry (LC–MS). The results indicate that peptides derived from goat milk fermented with *L. fermentum* exhibit antioxidant activity and that proteolytic activity can be optimized depending on the incubation time and inoculation rate. Studies have shown that the optimal fermentation time significantly affects both the microbial composition and the release of bioactive compounds [77]. Consequently, the optimal fermentation time determines the emergence of antioxidant properties and the extent to which these properties are present [84].

#### 3.1.3. Other Factors

In addition to these factors, fermentation efficiency can be affected by different conditions, including temperature and nutrient concentration [37,40,49]. Temperature is another factor that can influence fermentation. In the study by Therdtatha et al., coffee beans were fermented by various microorganisms [8]. It has been shown that maintaining a temperature of 30 °C during fermentation improves antioxidant activity and phenolic content [8]. Thermal treatment, a factor that impacts fermentation efficiency, accelerated the fermentation process of meads fermented with *Saccharomyces bayanus* or *S. cerevisiae* [39]. Thermal treatment is considered a beneficial technique for accelerating fermentation; however, it can pose safety risks due to its disadvantages [85]. Hydroxymethylfurfural (HMF), a compound produced during thermal processes, can lead to a decrease in the quality of products [86]. Higher levels of HMF can adversely affect the sensory properties and acceptability of the product [39,86]. Additionally, it may exhibit health risks as well as product properties. Therefore, the thermal treatment process should be carefully planned and controlled to mitigate these potential issues. Conversely, in the study conducted by Kwaw et al., the aim was to evaluate the effect of ultrasonication, pulsed light, and the combination of these two techniques on the phenolic concentration and antioxidant activity of mulberry juice fermented by LAB. As a result, the highest values were obtained when these two techniques were used together [87].

During the fermentation of milk with *L. delbrueckii* subsp. *acidophilus*, conventional heating, ultrasound, and microwave treatments were applied alone or in combination, and how these temperature applications affect postfermentation properties was investigated [85]. According to the results of the present study, although the peptide content increased in all the samples after fermentation, the ultrasound and microwave treatments increased the peptide content, possibly due to the increase in protein hydrolysis with these processes. According to the results of the ABTS and DPPH radical scavenging activity tests, the activity was greatest when ultrasonic and microwave treatments were performed together. Similarly, when ultrasonic and microwave treatments were performed together, the α-amylase inhibition activity and exopolysaccharide content were greater than those of the other combinations. The results of these studies revealed that different processes change the nutritional value and biological properties of products after fermentation [88,89].

On the other hand, processes such as ultrasonic treatment affect fermentation and postfermentation properties [16]. It was observed that different parameters altered the properties after fermentation. Therefore, the optimization of fermentation conditions contributes to and improves many characteristics of the final product [90]. By optimizing these parameters, it is possible to achieve the best antioxidant properties after fermentation, which can positively affect other functions. Since these parameters influence each other, the optimization process varies depending on the product.

## 4. Antioxidant Profiles of Fermented Food

Fermented foods are products that humans have consumed for centuries [15]. The primary purpose of fermentation is to improve the nutritional and functional properties of these products, particularly their preservation [12]. The fermentation process and the properties of the resulting product vary depending on the type of food being fermented [59]. Dairy products, including milk, yogurt, cheese, and kefir, as well as pickles, vinegar, kimchi, and kombucha, are widely consumed fermented foods that have been used throughout history [91]. Additionally, the fermentation of fruits, vegetables, and various other foods, including their byproducts or parts considered waste, has recently become widespread in the food industry [27].

### 4.1. Dairy Products

Milk and dairy products are widely consumed due to their nutritional properties [92,93]. Their high nutritional and significant protein content support nutrition, development, and overall health [94,95]. The multifunctional properties of the bioactive components of milk include anti-inflammatory, antiviral, neuroprotective, and immunomodulatory effects [96,97,98]. With increasing interest in healthy nutrition, the use of foods rich in bioactive content and the preservation and enhancement of bioactive content during the production of functional foods have attracted increasing amounts of attention [89]. Similarly, the widespread use of bioactive compounds with antiviral, antioxidant, and similar properties as food and supplements has become common [99,100]. Recently, there has been increasing interest in a significant milk bioactive component known as lactoferrin, which has distinctive properties, including virus inhibition, such as in COVID-19 [101,102]. The benefits they provide are important for the health of mothers, infants, children, adolescents, adults, elderly people, and athletes [14,103].

Diverse animal milks are widely used to obtain dairy products, including fermented milk, kefir, yogurt, and cheese, due to their various bioactive components and nutritional value [62,104]. At this point, milk from different animals, especially bovine, buffalo, goat, and camel milk, is commonly utilized [88,105,106]. The main reasons for this are their nutritional value, bioactive components, health benefits, and taste and flavor diversity [85,107]. In a study conducted by Ayyash et al., camel milk and bovine milk were fermented by *Lactobacillus* spp. [108]. As a result of their fermentation, α-amylase and α-glucosidase inhibition, angiotensin-converting enzyme (ACE) inhibition, and antioxidant, proteolytic, and anticancer activities were evaluated. It was observed that camel milk exhibits greater proteolytic activity than bovine milk. Both types of milk fermented with all the strains exhibited α-amylase and α-glucosidase inhibition activity. When the effects of ACE inhibition were evaluated, the effects of camel milk significantly increased compared to those of bovine milk during the storage period. The ability of fermented cattle and camel milk to inhibit the proliferation of Caco-2, MCF-7, and HeLa cancer cell lines was evaluated, and it was revealed that camel milk exhibited greater inhibitory effects than bovine milk. Compared with fermented bovine milk, fermented camel milk exhibited greater antioxidant activity, as indicated by higher ABTS scavenging rates and DPPH scavenging rates.

The chemical, microbiological, and biological properties of kefir produced from sheep’s milk were investigated in this study [58]. Significant changes were not observed in the protein, fat, total carbohydrate, caloric, ash, moisture, or mineral contents of the products. However, the lactose content notably decreased after fermentation. Furthermore, peptide signals of fermented products were analyzed using matrix-assisted laser desorption/ionization time-of-flight mass spectrometry (MALDI-TOF-MS), revealing an increased number of peptide signals in sheep’s milk fermented with kefir grains.

Additionally, products developed by enriching different plants and plant extracts to milk and dairy products tend to exhibit greater antioxidant activity [109,110]. In a study aiming to determine the effect of moringa extract on the fermentation process, quality, and bioactivity of yogurt, fermentation was achieved using starter cultures of *Bifidobacterium longum* subsp. *longum*, *Streptococcus salivarius* subsp. *thermophilus*, and *L. delbrueckii* subsp. *acidophilus* [111]. Moringa extract at different concentrations increases the fermentation rate and improves the phenolic content in a concentration-dependent manner. Similarly, with increasing moringa concentration, the cellular H_2_O_2_ concentration in the human colorectal cell line HT-29 decreased significantly. In another study, the effects of adding pomegranate peel polyphenols to the fermentation of milk containing *L. delbrueckii* subsp. *acidophilus* were investigated [22]. The total polyphenol content increased after fermentation, and the extract exhibited DPPH radical scavenging activity. The addition of multifaceted bioactive compounds such as polyphenols during fermentation enriches the product content and enhances its antioxidant properties [112,113]. Similarly, Ge et al. reported that the addition of sea buckthorn increased the total phenolic and flavonoid contents of milk fermented with various LAB species [114]. On the other hand, antioxidant activity was determined by DPPH radical scavenging activity, reducing antioxidant power (FRAP), and Fe_3+_ reducing power analyses. The antioxidant activity of milk samples fermented with different LAB species improved significantly.

In a study aimed at producing and storing fermented skim milk fortified with chestnut flour, the research sought to ascertain the impact of chestnut flour supplementation on the antioxidant and physicochemical attributes of the final product [115]. In this study, *L. delbrueckii* subsp. *acidophilus*, *Lacticaseibacillus rhamnosus*, and *Bifidobacterium animalis* ssp. *lactis* (*B. animalis* ssp. *lactis*) were used during fermentation. It was observed that samples fermented with *B. animalis* ssp. *lactis* exhibited the highest phenolic content. Conversely, the antioxidant capacity was assessed through Trolox equivalent antioxidant capacity (TEAC), DPPH, and FRAP methods, revealing that samples fermented with *L. delbrueckii* subsp. *acidophilus* demonstrated the highest antioxidative potential. Furthermore, the inclusion of chestnut flour positively influenced the stabilization of the products and mitigated the rate of syneresis. Thus, the incorporation of chestnut flour enhanced both the functional and nutritional aspects of the fermented milk.

The fermentation of milk and dairy products enhances their bioactive content, enabling them to exhibit antioxidant properties (Table 1) [116,117]. Furthermore, it was noted that adding different products during fermentation can enhance the nutritional and functional properties of these products [118,119].

### 4.2. Other Products

In addition to dairy products, many fermented foods are traditionally consumed worldwide. These fermented products are typically plant- or algae-based and can include various products, such as beverages and sauces (Table 2) [122,123]. In particular, the consumption of plant-based fermented milk and dairy products is increasing globally, offering an alternative for individuals who do not consume or cannot consume animal milk [124]. Additionally, many plant-based food products exhibit enhanced nutritional and functional properties through fermentation, resulting in a wide range of innovative products, including beverages, sauces, and grain products [125,126].

The use of milk obtained from different plant-based materials, especially yogurt, has increased in recent years [127,128]. In a recent article, the physicochemical properties, antioxidant activity, and sensory characteristics of peanut milk-based yogurt containing *Lactobacillus* spp. were examined after fermentation [129]. That study aimed to assess the effects of fermentation on these parameters. The results indicated an increase in phenolic and flavonoid contents. Antioxidant activity was evaluated using three different methods: DPPH, fractional inhibitory concentration (FIC), and FRAP. The yogurt exhibited the highest DPPH scavenging activity when it was prepared with *L. rhamnosus*. Additionally, a significant increase in FIC activity was observed for all products. Overall, the study demonstrated that probiotic bacteria had a positive impact on the physicochemical properties and antioxidant activity of peanut milk-based yogurt. In another study, the outcomes of fermenting soy milk with three different LAB *strains—L. plantarum*, *Levilactobacillus brevis*, and *Limosilactobacillus reuteri*—were investigated [130]. Antioxidant activity varied depending on the bacterial strain used; notably, samples fermented with *L. plantarum* showed a significant increase in antioxidant activity. Additionally, an increase in oligosaccharides during the fermentation process was observed. These findings underscore the potential of lactic acid bacteria fermentation to enhance the nutritional value and health benefits of plant-based milk alternatives.

The fermentation of plant material, including vegetables, fruits, and leaves, commonly enhances the bioactive components of these parts [131]. For example, in a study investigating the utilization of avocado leaves to enrich phenolic contents through fermentation, De Montijo-Prieto et al. demonstrated the strain-specific metabolism of phenolic compounds using various LAB strains [76]. The quantity of phenolic compounds varied depending on the strain employed. Fermentation affects phenolic content, with variations observed across different strains [76]. Additionally, this process facilitates the release of phenolic compounds, thereby increasing the bioavailability of phenols through enzymatic activity. Among the LAB strains utilized in fermentation, *Pediococcus pentosaceus* exhibited the most significant increase in phenolic content according to De Montijo-Prieto et al. [76]. In another study with garlic, it was reported that the nutritional composition of garlic improves after fermentation; in particular, its mineral profile and phenolic content are affected by fermentation [132]. Additionally, a series of analyses revealed increased antioxidant properties. It was demonstrated that fermentation enhances the preservation and shelf life of this food. A recent study aimed to investigate the physicochemical and biological properties of polysaccharides obtained from okra (*Abelmoschus esculentus*) after fermentation using various *L. plantarum* strains [133]. Specifically, the effects of fermentation on the chemical composition, molecular weight, monosaccharide composition, viscosity, structural properties, and antioxidant and immunomodulatory activities of polysaccharides were examined. The results revealed that polysaccharides derived from okra showed differences in monosaccharide composition following fermentation, with particularly significant changes in glucose, galactose, and mannose ratios. Additionally, a noticeable reduction in viscosity was observed after fermentation, indicating that the fermentation process influenced the molecular structure and water-binding capacity of the polysaccharides. Fourier transform infrared spectroscopy (FT-IR) analysis confirmed that fermentation altered the chemical bonds and functional groups of polysaccharides, thus modifying their structural characteristics. For the assessment of immunomodulatory activity, cytokine measurements and immunoregulatory gene expression analyses were conducted. Compared with nonfermented polysaccharides, fermented polysaccharides significantly enhanced macrophage phagocytic activity and cytokine production. Antioxidant activity was evaluated using three different methods: DPPH, ABTS, and FRAP. Similarly, compared with nonfermented polysaccharides, fermented polysaccharides exhibited greater DPPH and ABTS radical scavenging activities. Likewise, FRAP analysis indicated that the fermented polysaccharides had a significantly greater iron-reducing capacity. Additionally, according to Zhang et al., the antioxidant activity of tea depends on the degree of fermentation, which can alter the scavenging activity of Pu-erh tea [57].

Fermentation is an effective method for enhancing polyphenol content, increasing antioxidant properties, and improving the bioavailability of fruit and vegetable juices [46,73]. One study involved the fermentation of a beverage made with cupuassu, a tropical fruit native to Brazil, using *Lactobacillus casei*. During fermentation, the sugars in the cupuassu, including fructose, sucrose, and glucose, were consumed, especially fructose. Citric, ascorbic, and quinic acids, among the organic acids found in cupuassu, promoted the growth of *L. casei* and were consumed during fermentation, thereby increasing the nutritional value of the drink [134]. In a study conducted by Suazo et al., the type of fermentation and the microorganisms used were not clearly mentioned; however, the results showed that the phenolic content of fermented cocoa beans was dramatically lower than that of nonfermented cocoa beans [135]. In this case, the effect of fermentation may differ in the fermented product.

The use of seaweeds as food and food ingredients has attracted attention in recent years, especially because of their bioactive content and benefits for human health [15,136]. A sauce was made after fermenting *P. yezoensis* algal species with *L. plantarum* and *L. casei* [74]. LAB fermentation continued for 72 h. An increase in the lactic acid content resulted in the conversion of phenolic compounds in the sauce. It was also shown that fermentation increased the production of volatile compounds and improved the taste and sensory properties of the sauce.

**Table 2:** Impact of fermentation type and used microorganisms on antioxidant activity in food products.

Categories of Product	Fermented Product	Fermentation Type	Fermented by	Outcome	References
Plant-based milk and milk products	Oat and soy milk	Lactic acid bacteria fermentation	*Lactiplantibacillus plantarum*	-Exhibit radical scavenging activity	[124]
	Cashew milk-based yogurt	Lactic acid bacteria fermentation	*Lacticaseibacillus rhamnosus* *Lacticaseibacillus casei* *Lactiplantibacillus plantarum*	-Increase phenolic content -Increase flavonoid content	[129]
	Chickpea yam milk	Lactic acid bacteria fermentation	*Lacticaseibacillus rhamnosus*	-Exhibit radical scavenging activity	[12]
	Soymilk	Lactic acid bacteria fermentation	*Lactiplantibacillus plantarum* *Levilactobacillus brevis* *Limosilactobacillus reuteri*	-Increase phenolic content	[130]
	Soymilk	Lactic acid bacteria fermentation	*Lactiplantibacillus plantarum*	-Exhibit an increasing β-galactosidase activity -Exhibit radical scavenging activity	[127]
	Rice milk	Lactic acid bacteria fermentation	Lactic acid bacteria	-Exhibit radical scavenging activity	[26]
	Hickory yogurt	*	*Lactobacillus delbrueckii* subsp. *bulgaricus* *Streptococcus salivarius* subsp. *thermophilus*	-Exhibit radical scavenging activity	[128]
	Sesame milk	Lactic acid bacteria fermentation	*Lactiplantibacillus plantarum*	-Exhibit an increasing β-galactosidase activity	[125]
Plant samples, vegetables, and fruits	Lvjian okra	Lactic acid bacteria fermentation	*Lactiplantibacillus plantarum*	-Exhibit radical scavenging activity -Exhibit ferric reducing power	[133]
	Avocado leaf extracts	Lactic acid bacteria fermentation	*Pediococcus acidilactici* *Pediococcus pentosaceus* *Leuconostoc mesenteroides* subsp. *mesenteroides* *Levilactobacillus brevis* *Lactiplantibacillus plantarum* subsp. *plantarum* *Lactiplantibacillus plantarum*	-Increase phenolic content	[76]
	Coix seed	*	*Saccharomyces cerevisiae*	-Exhibit radical scavenging activity	[137]
	Dark tea	Solid-state fermentation	*Bacillus subtilis*	-Altering catechin amount	[53]
	*Coprinus comatus*	Liquid fermentation	*	-Exhibit scavenging activity	[80]
	Corn bran	Solid-state fermentation	*Lactiplantibacillus plantarum* *Limosilactobacillus reuteri*	-Increase phenolic content	[54]
	Coffee beans	Solid-state fermentation	*Hanseniaspora osmophila* *Hanseniaspora Vineae* *Schizosaccharomyces osmophilus* *Lactiplantibacillus plantarum*	-Increase phenolic content	[8]
	Garlic	*	*	-Increase phenolic content -Increase flavonoid content	[132]
	Green tea	Lactic acid bacteria fermentation followed by acetic acid fermentation	*Acetobacter pasteurianus* *Lacticaseibacillus paracasei* *Saccharomyces cerevisiae*	-Exhibit radical scavenging activity	[90]
	Wheat bran	Solid-state fermentation	*Lactiplantibacillus plantarum* *Saccharomyces cerevisiae*	-Increase phenolic content	[50]
	Pollen	Solid-state fermentation	*Lactobacillus rhamnosus*	-Increase phenolic content -Increase flavonoids content -Exhibit scavenging activity	[51]
	*Angelica pubescens*	Submerged fermentation	*	-Exhibit scavenging activity	[30]
	*Pogostemon cablin*				
	*Paeonia lactiflora*				
	*Alpinia oxyphylla*				
	*Melaleuca leucadendron*				
	*Osmanthus fragrans*				
	*Glycyrrhiza uralensis*				
	*Phellodendron chinense*				
	Rice				
	Edible grass	Lactic acid bacteria fermentation	*Lactiplantibacillus plantarum* *Lactobacillus rhamnosus*	-Exhibit scavenging activity	[49]
	Chestnut inner shell	Alcohol fermentation	*Aspergillus sojae*	-Improve bioactive components	[32]
	Barley grain	*	*Lactiplantibacillus plantarum*	-Improve bioactive components -Exhibit scavenging activity	[122]
	Wheat sourdough	Lactic acid bacteria fermentation	*Lactiplantibacillus plantarum* *Lactobacillus casei*	-Increase polyphenol content	[79]
	African nightshade leaves	Lactic acid bacteria fermentation	*Lactiplantibacillus plantarum* *Weissella cibaria* *Leuconostoc pseudomesenteroides*	-Increase phenolic content -Exhibit radical scavenging activity	[42]
	Dandelion (*Taraxacum officinale*)	Solid-state fermentation	*Lactiplantibacillus plantarum* *Saccharomyces cerevisiae*	-Increase flavonoid content	[1]
	Soybean flour	Solid-state fermentation	*Lactobacillus casei*	-Exhibit an improvement of nutritive value -Increase flavonoids content -Exhibit an increase flavonoids metabolite -Exhibit scavenging activity	[55]
	Rice bran	Lactic acid bacteria fermentation	*Lactiplantibacillus plantarum*	-Exhibit scavenging activity	[71]
	Wheat bran				
	*Cyperus rotundus* L.	Lactic acid bacteria fermentation	*Lactiplantibacillus plantarum*	-Exhibit radical scavenging activity	[131]
	*Lavandula angustifolia* extract	*	*Pediococcus pentosaceus*	-Inhibit ROS generation	[10]
	Soybean	*	*Lactobacillus delbrueckii* subsp. *bulgaricus* *Streptococcus salivarius* subsp. *thermophilus*	-Exhibit radical scavenging activity	[6]
	Green coffee beans	*	*Saccharomyces cerevisiae* *Saccharomycopsis fibuligera*	-Increase flavonoid content	[4]
	Kombucha tea	*	*	-Improve bioactive content -Exhibit radical scavenging activity	[123]
	Black rice bran	Solid-state fermentation	*Aspergillus awamori* *Aspergillus oryzae*	-Increase phenolic content	[7]
	Rice bran	Lactic acid bacteria fermentation	*Lactococcus lactis* *Lactiplantibacillus plantarum*	-Increase phenolic content -Exhibit radical scavenging activity	[77]
	*Diospyros lotus* fruit	*	*Microbacterium flavum* *Lactiplantibacillus plantarum*	-Exhibit radical scavenging activity -Exhibit an inhibitory effect on α-glucosidase activities	[126]
	Rice flour and black gram flour	Solid-state fermentation	Yeast	-Increase phenolic content -Improve increase bioactive content	[5]
	*Lablab purpureus*	Solid-state fermentation	*Aspergillus oryzae* *Aspergillus awamori*	-Increase phenolic content -Exhibit α-amylase activity	[21]
	Rice bran	Solid-state fermentation	*Aspergillus oryzae* *Rhizopus oryzae*	-Exhibit radical scavenging activity	[33]
	Quinoa seeds	*	*Saccharomyces cerevisiae*	-Increase phenolic content	[68]
	Myrtle (*Myrtus communis*) berries	Lactic acid bacteria fermentation	*Lactiplantibacillus plantarum*	-Exhibit radical scavenging activity -Increase phenolic content	[138]
	Red cabbage	Lactic acid bacteria fermentation	*Lactiplantibacillus plantarum* *Lactobacillus delbrueckii* subsp. *acidophilus*	-Increase phenolic content	[29]
	Pu-erh tea	Pile-fermentation	*	-Exhibit scavenging activity	[57]
Beverage	Mead	Alcohol fermentation	*Saccharomyces bayanus* *Saccharomyces cerevisiae*	-Exhibit scavenging activity	[39]
	Pomegranate juice	Lactic acid bacteria fermentation	*Lactiplantibacillus plantarum*	-Increase phenolic content	[73]
	Goji juice	*	*Bacillus velezensis* *Bacillus licheniformis* *Limosilactobacillus reuteri* *Lacticaseibacillus rhamnosus* *Lactiplantibacillus plantarum*	-Increase phenolic content -Exhibit scavenging activity	[139]
	Pomegranate juice	Lactic acid bacteria fermentation	*Lactiplantibacillus plantarum* *Lactobacillus delbrueckii* subsp. *acidophilus*	-Exhibit scavenging activity -Increase phenolic content	[9]
	Apple juice	Lactic acid bacteria fermentation	*Lactiplantibacillus plantarum*	-Exhibit scavenging activity	[28]
	Pear juice	Lactic acid bacteria fermentation	*Lactiplantibacillus plantarum* *Lactobacillus helveticus* *Lacticaseibacillus casei*	-Increase phenolic content -Improve the formation of alcohols, esters, acids, and terpenoids -Reduce the content of aldehydes and ketones -Exhibit scavenging activity	[44]
	Strawberry juice	Lactic acid bacteria fermentation	*Lactiplantibacillus plantarum* *Lactobacillus delbrueckii* subsp. *acidophilus*	-Increase phenolic content -Exhibit scavenging activity	[70]
	Wolfberry and longan juice	Lactic acid bacteria fermentation	*Lacticaseibacillus paracasei* *Lactococcus lactis subsp. lactis*	-Alter metabolite profile	[46]
	Cupuassu	Lactic acid bacteria fermentation	*Lactobacillus casei*	-Increase phenolic content	[134]
	*Chamerion angustifolium*	Solid-state fermentation	*	-Increase flavonoid content	[81]
	Mulberry juice	Lactic acid bacteria fermentation	*Lactiplantibacillus plantarum*	-Increase phenolic content -Increase flavonoid content -Increase anthocyanin content	[87]
		Lactic acid bacteria fermentation	*Lactiplantibacillus plantarum* *Lactobacillus delbrueckii* subsp. *acidophilus* *Lacticaseibacillus paracasei*	-Increase phenolic content -Increase flavonoid content -Increase anthocyanin content -Exhibit scavenging activity	[72]
	Murta (*Ugni molinae*) juice	Lactic acid bacteria fermentation	*Leuconostoc mesenteroides*	-Increase phenolic content -Improve bioactive content -Exhibit scavenging activity	[20]
	Apple juice	Lactic acid bacteria fermentation	*Lactiplantibacillus plantarum* *Lactobacillus helveticus* *Lacticaseibacillus casei* *Lactobacillus delbrueckii* subsp. *acidophilus* *Lacticaseibacillus paracasei* *Bifidobacterium lactis*	-Exhibit scavenging activity	[45]
	Goji juice	Lactic acid bacteria fermentation	*Lacticaseibacillus paracasei* *Lacticaseibacillus rhamnosus* *Lactiplantibacillus plantarum*	-Exhibit scavenging activity	[31]
	Kombucha beverage	*	Symbiotic cultures of bacteria and yeasts	-Increase polyphenol content	[82]
	Strawberry beverage	Gluconic fermentation	*Gephyroberyx japonicus*	-Increase cell viability -Reduce oxidative stress	[3]
		Alcohol fermentation	*Saccharomyces cerevisiae*		
	Cabernet sauvignon wine	Mixed fermentation	*Pichia kudriavzevii* *Saccharomyces cerevisiae*	-Increase phenolic content	[60]
Sauce	*Porphyra yezoensis*	Lactic acid bacteria fermentation	*Lactiplantibacillus plantarum* *Lacticaseibacillus casei*	-Increase free amino acids	[140]
		Lactic acid bacteria fermentation	*Lactobacillus fermentum* *Lactobacillus casei* *Streptococcus salivarius* subsp. *thermophilus*	-Exhibit scavenging activity	[74]
	*Himanthalia elongata*	Solid-state fermentation	*Lactobacillus casei* *Lacticaseibacillus paracasei* *Lactobacillus rhamnosus* *Bacillus subtilis*	-Decrease phenolic content	[136]

* Not identified.

### 4.3. Waste and Byproducts

The valorization of food waste and byproducts within the framework of zero waste policies has become a significant issue in recent years [61]. In particular, the utilization of food waste or byproducts considered waste through fermentation for nutraceutical or functional purposes is seen as a crucial step in supporting zero waste policies [27,141]. By enhancing the antioxidant activities of fruit, vegetable, beverage, and fish byproducts, which are considered waste, as well as fermented foods, through the fermentation process, the aim is to provide both environmental and economic benefits, particularly through the utilization of industrial and agro-industrial byproducts and wastes (Table 3) [120,142].

In a study conducted by Kaur et al., the effect of different parameters (pH, incubation temperature, nitrogen source, and incubation time) on β-carotene production was examined through solid-state fermentation using three fruit and vegetable wastes, namely, orange, carrot, and papaya, with *Blakeslea trispora*. In that study, three different antioxidant tests were applied: DPPH, ABTS, and FRAP [40]. The results confirmed that β-carotene produced from *Blakeslea trispora* has high antioxidant activity. These findings suggest that β-carotene can effectively neutralize free radicals and can be considered a potential source of natural antioxidants [40].

The effect of adding mango peel during the submerged fermentation of milk with LAB was investigated [84]. The pH reductions occurred in both the control and mango peel samples. This change is thought to result from the lactic acid produced by LAB and the bioconversion of polysaccharides in the mango peel into glucose. This pH reduction was shown to contribute to the dissolution of phenolic compounds in mango peels, thereby promoting antioxidant activity. When evaluating antioxidant properties, it was determined that fermentation with added mango peel exhibited greater antioxidant activity in the DPPH radical scavenging activity and FRAP assays. On the other hand, no significant difference was found in the antioxidant activities measured by the ABTS assay. Another study on solid-state fermentation using *Aspergillus oryzae* on rice bran byproducts evaluated changes in bioactive compounds and antioxidant properties [143]. The results indicated that fermentation increased the bioactive components of the rice bran byproduct. Significant enhancements in antioxidant properties were observed in the evaluations of DPPH and ABTS+ radical scavenging activities.

Additionally, some drying methods are also used to preserve the bioactive content of these wastes and byproducts. In a recent study, the effects of freeze-dried apple pomace and pomegranate peel powders on probiotic yogurt were investigated [144]. The addition of these powders increased the total phenolic content and antioxidant capacity of the yogurt.

**Table 3:** Valorization of waste and byproducts as an antioxidant source by fermentation.

Wastes and Byproducts	Fermentation Type	Fermented by	Outcome	References
Cauliflower byproducts	Lactic acid bacteria fermentation	*Levilactobacillus brevis* *Lactiplantibacillus plantarum*	-Exhibit scavenging activity	[141]
*Siraitia grosvenorii* pomace	Solid-state fermentation	*Eurotium cristatum*	-Increase phenolic content	[142]
Apple pomace and pomegranate peel powders	*	*Lactobacillus bulgaricus* *Streptococcus salivarius* subsp. *thermophilus*	-Increase phenolic content	[144]
Kiwifruit pulp	Lactic acid bacteria fermentation	*Lactiplantibacillus plantarum*	-Increase phenolic content -Increase flavonoid content -Exhibit scavenging activity -Reduce oxidative stress	[2]
Sea bass samples	Lactic acid bacteria fermentation	*Lactiplantibacillus plantarum*	-Increase phenolic content	[48]
Rice bran byproduct	Solid-state fermentation	*Aspergillus oryzae*	-Improve bioactive components -Exhibit scavenging activity	[143]
Apple pomace	Solid-state fermentation	*Phanerochaete chrysosporium*	-Increase phenolic content	[11]
Red bayberry pomace	Microbial fermentation	Yeast powder	-Increase flavonoid content	[59]
	Lactic acid bacteria fermentation	Yeast powder and lactic acid bacteria powder		
	Alcohol fermentation	Yeast powder and acetic bacteria		
	Microbial fermentation	Yeast powder, lactic acid bacteria powder, and acetic bacteria		
Fruit and vegetable wastes	Solid-state fermentation	*Blakeslea trispora*	-Increase β-carotene production -Exhibit scavenging activity	[40]
Orange pomace	Solid-state fermentation	*Paecilomyces variotii*	-Increase phenolic content	[27]
*Prunus armeniaca* L. pomace	Solid-state fermentation	*Aspergillus niger* *Rhizopus oligosporus*	-Increase phenolic content -Exhibit scavenging activity	[61]

* Not identified.

The use of natural antioxidants in the food industry, preventive medicine, food, cosmetics, and pharmaceutical products has recently become widespread [126,138]. Significantly, the anti-aging agents derived from plants and plant materials are preferred products composed of natural compounds [137]. As an antioxidant source, the fermentation of dairy products, plant-based products, waste, and byproducts improves the bioactive components of these materials, which can enhance general health [61].

## 5. Conclusions

In this article, the effects of different types of fermentation on the antioxidant activities of food products were examined, revealing that optimization yields varying results for each product. Fermentation not only preserves but also enhances the nutritional and functional properties of foods. Particularly, in the bioconversion of products, the phenolic content significantly increases, thereby strengthening the antioxidant properties. Additionally, the radical scavenging activity of food is almost always enhanced after fermentation. Fermentation can also improve other properties, including the flavor and texture of products.

In the dairy industry, fermented products have the potential to extend shelf life and attract attention due to their rich bioactive content. Recently, the addition of fermented plant-based materials to dairy products has proven effective in enhancing functional and nutritional properties, including antioxidant activity. These products also offer solutions for individuals who do not consume dairy products. Moreover, research on the fermentation of food waste and the valorization of food products under zero waste policies is gaining momentum. This approach not only improves waste management but also promotes the production of antioxidant-rich food products. In conclusion, fermentation has emerged as an effective method for enhancing the antioxidant activity of food products. New studies are needed to optimize and prove these effects for various industries, including the food industry. Examining not only the antioxidant properties of food products but also how these properties change after fermentation and determining the relationship between the consumption of these products and their health benefits will provide valuable direction for future research.

## Figures and Tables

**Table 1:** Correlation between fermentation and antioxidant activity in dairy products.

Fermented Product	Fermentation Type	Fermented by	Outcomes	Concentration of Activities		References
Buffalo yogurt	Lactic acid bacteria fermentation	*Streptococcus salivarius* subsp. *thermophilus* *Lactobacillus delbrueckii* subsp. *bulgaricus*	-Exhibit radical scavenging activity -Exhibit angiotensin I-converting enzyme inhibitory activity	Radical scavenging activity (mg TE 100 g^−1^)	Angiotensin I-converting enzyme inhibitory activity (%)	[86]
				7.06 ± 0.04	28.82 ± 0.04	
Goat yogurt				8.18 ± 0.05	34.69 ± 0.04	
Sheep yogurt				9.34 ± 0.02	38.51 ± 0.08	
Milk	Lactic acid bacteria fermentation	*Lactobacillus pentosus* *Limosilactobacillus fermentum* *Lacticaseibacillus paracasei* subsp. *tolerans*	-Exhibit radical scavenging activity	12.7–16.3%		[114]
			-Increase total phenolic content	36–97 μg mL^−1^		
			-Increase total flavonoid content	0.54–0.55 mg RU mL^−1^		
Horse milk	*	*Lactobacillus bulgaricus* *Streptococcus salivarius* subsp. *thermophilus*	-Exhibit radical scavenging activity	63.04 ± 6.85%		[24]
Camel milk	Lactic acid bacteria fermentation	*Lactobacillus helveticus* *Lacticaseibacillus casei* subsp. *casei* *Lacticaseibacillus paracasei* *Lacticaseibacillus rhamnosus*	-Exhibit radical scavenging activity	0.322 mg GAE mL^−1^		[64]
Milk	*	*Lactobacillus* *delbrueckii* subsp. *bulgaricus* *Streptococcus salivarius* subsp. *thermophilus*	-Increase phenolic content	89.25–532.24 mg GAE L^−1^		[120]
Milk	*	*Lactiplantibacillus plantarum* *Bifidobacterium animalis* ssp. *lactis* *Streptococcus salivarius* subsp. *thermophilus*	-Exhibit radical scavenging activity	90.35–95.62%		[75]
Kefir	Submerged fermentation	Lactic acid bacteria	-Exhibit radical scavenging activity			[84]
			-Exhibit total reducing capacity	*		
Milk	Lactic acid bacteria fermentation	*Lactobacillus delbrueckii* subsp. *acidophilus*	-Exhibit radical scavenging activity	64.7%		[85]
			-Exhibit an inhibitory effect on α-amylase	*		
			-Increase exopolysaccharide content	*		
Goat milk	Lactic acid bacteria fermentation	*Lactiplantibacillus plantarum*	-Exhibit an inhibitory effect on α-amylase and α-glucosidase activities -Increase exopolysaccharide content	*		[88]
Milk	Lactic acid bacteria fermentation	*Lactobacillus helveticus* *Limosilactobacillus reuteri* *Lacticaseibacillus rhamnosus*	-Exhibit radical scavenging activity	415–1045 µM TE		[66]
			-Exhibit angiotensin I-converting enzyme inhibitory activity	0.18–2.63 mg mL^−1^		
Milk	*	*Streptococcus salivarius* subsp. *thermophilus* *Lactobacillus delbrueckii* subsp. *bulgaricus* *L. delbrueckii* subsp. *Acidophilus*	-Exhibit radical scavenging activity	2.16–24.44%		[65]
Bovine colostrum	*	*Candida lipolytica*	-Exhibit radical scavenging activity	63–92%		[89]
			-Exhibit angiotensin I-converting enzyme inhibitory activity	72.85–78.52%		
Goat milk	Lactic acid bacteria fermentation	*Lactobacillus fermentum*	-Exhibit radical scavenging activity	0.32–55.73%		[83]
			-Exhibit higher proteolytic activity	1.57–8.44 mg mL^−1^		
Yogurt	Lactic acid bacteria fermentation	*Streptococcus salivarius* subsp. *thermophilus* *Lactobacillus delbrueckii* subsp. *acidophilus* *Bifidobacterium longum* subsp. *longum*	-Increase phenolic content	*		[111]
			-Exhibit radical scavenging activity	73.32% in DPPH and 86.29% in ABTS assays		
Milk	Lactic acid bacteria fermentation	*Lactiplantibacillus plantarum*	-Exhibit radical scavenging activity	*		[16]
Milk	Lactic acid bacteria fermentation	*Lacticaseibacillus casei*	-Exhibit angiotensin I-converting enzyme inhibitory activity	42.78–52.28%		[43]
Skim camel milk	Lactic acid bacteria fermentation	*Limosilactobacillus reuteri* *Lactiplantibacillus plantarum*	-Exhibit radical scavenging activity	30–70%		[108]
			-Exhibit angiotensin I-converting enzyme inhibitory activity	*		
Milk	Lactic acid bacteria fermentation	*Lactobacillus delbrueckii* subsp. *acidophilus*	-Increase phenolic content	2.6–6.8 GAE mL^−1^		[22]
			-Exhibit radical scavenging activity	23–42 μmol TE mL^−1^		
Camel milk	Lactic acid bacteria fermentation	*Lacticaseibacillus lactis* *Lactobacillus delbrueckii* subsp. *acidophilus*	-Exhibit an inhibitory effect on α-amylase and α-glucosidase activities	*		[121]
			-Exhibit angiotensin I-converting enzyme inhibitory activity	*		
			-Exhibit radical scavenging activity	30–50%		
Goat milk	Lactic acid bacteria fermentation	*Lacticaseibacillus casei*	-Exhibit radical scavenging activity	56.50–88.01%		[25]
Yogurt	Lactic acid bacteria fermentation	*Lactobacillus delbrueckii* subsp. *acidophilus* *Bifidobacterium longum* subsp. *longum* *Streptococcus salivarius* subsp. *thermophilus*	-Exhibit radical scavenging activity	*		[109]
Kefir	Microbial fermentation	Lactic acid bacteria Yeast	-Exhibit radical scavenging activity	*		[58]
Milk	*	*Lactobacillus delbrueckii* subsp. *acidophilus*	-Exhibit ferric reducing power	22.24 mg AE 100 g^−1^		[118]
Buffalo yogurt	Lactic acid bacteria fermentation	*Lactobacillus delbrueckii* subsp. *acidophilus* *Lactobacillus helveticus* *Lactobacillus delbrueckii* subsp. *bulgaricus* *Streptococcus salivarius* subsp. *thermophilus*	-Exhibit radical scavenging activity	70.14–81.62%		[47]
Milk	Lactic acid bacteria fermentation	*Streptococcus salivarius* subsp. *thermophilus* *Lactobacillus delbrueckii* subsp. *acidophilus* *Bifidobacterium animalis* spp. *lactis*	-Increase phenolic content	333–2409 mg GAE L^−1^		[113]
Sarshir	Lactic acid bacteria fermentation	*Lactiplantibacillus plantarum*	-Exhibit radical scavenging activity	53.1 ± 1.8%		[91]
Goat milk	Lactic acid bacteria fermentation	*Lactiplantibacillus plantarum*	-Exhibit angiotensin I-converting enzyme inhibitory activity -Exhibit radical scavenging activity	*		[105]
Yogurt	Lactic acid bacteria fermentation	*Streptococcus salivarius* subsp. *thermophilus* *Lactobacillus delbrueckii* subsp. *bulgaricus*	-Increase phenolic content	0.165–0.223 mg GAE mL^−1^		[110]
			-Exhibit radical scavenging activity	80.23–95.56%		
			-Exhibit metal chelating activity	95.88–96.75%		
Milk	*	*Lactobacillus delbrueckii* subsp. *acidophilus* *Lacticaseibacillus rhamnosus* *Bifidobacterium animalis* ssp. *lactis*	-Increase phenolic content	51.403–62.367 mg GAE 100 g^−1^		[115]
			-Exhibit radical scavenging activity	12.759–13.312 mg TE 100 mL^−1^		
Yak yogurt	Spontaneous fermentation	*	-Exhibit radical scavenging activity	*		[63]
Yogurt	Lactic acid bacteria fermentation	*Lactobacillus delbrueckii* subsp. *bulgaricus* *Lactobacillus delbrueckii* subsp. *acidophilus* *Streptococcus salivarius* subsp. *thermophilus*	-Exhibit an increasing β-galactosidase activity	0.13–0.19 U mL^−1^		[116]
			-Increase phenolic content	1.23–3.26 mg mL^−1^		
			-Exhibit radical scavenging activity	24–63%		
			-Exhibit metal chelating activity	*		
Milk	Lactic acid bacteria fermentation	Lactic acid bacteria	-Exhibit angiotensin I-converting enzyme inhibitory activity	26.31–75.87%		[117]
			-Exhibit radical scavenging activity	42.78–83.57%		
			-Exhibit metal chelating activity	*		
Milk and yogurt	Lactic acid bacteria fermentation	*Streptococcus salivarius* subsp. *thermophilus* *Lactobacillus delbrueckii* subsp. *bulgaricus* *Bifidobacterium animalis* ssp. *lactis*	-Exhibit radical scavenging activity	*		[62]
Bovine skim milk	*	*Lactobacillus helveticus*	-Exhibit radical scavenging activity	41.34–48.01%		[13]
			-Exhibit proteolytic activity	*		
Yogurt	Lactic acid bacteria fermentation	*Lactobacillus delbrueckii* subsp. *acidophilus* *Bifidobacterium animalis* ssp. *lactis* *Lacticaseibacillus casei* *Streptococcus salivarius* subsp. *thermophilus* *Lactobacillus delbrueckii* subsp. *bulgaricus*	-Increase phenolic content	2.811–3.220 mg GAE mL^−1^		[23]
			-Exhibit radical scavenging activity	97.71–98.53%		
Bovine milk	Lactic acid bacteria fermentation	*Lacticaseibacillus casei* *Lacticaseibacillus rhamnosus*	-Exhibit angiotensin I-converting enzyme inhibitory activity	48–100%		[67]
			-Exhibit radical scavenging activity	*		
Donkey milk	Lactic acid bacteria fermentation	*Streptococcus salivarius* subsp. *thermophilus* *Lactobacillus delbrueckii* subsp. *bulgaricus*	-Exhibit radical scavenging activity	63.38–66.21%		[107]
Kefir	*	*Saccharomyces cerevisiae* *Kazachstania exigua* *Acetobacter okinawensis* *Leuconostoc pseudomesenteroides* *Lactococcus lactis subsp. Lactis*	-Exhibit radical scavenging activity	1.22 mg mL^−1^		[41]
Kefir	Lactic acid bacteria fermentation	*	-Increase phenolic content	266.62–1232.33 mg GAE mL^−1^		[106]
			-Exhibit radical scavenging activity	15.68 μmol TE mL^−1^		
Yogurt	Lactic acid bacteria fermentation	*Lactobacillus delbrueckii* subsp. *bulgaricus* *Lacticaseibacillus casei* *Streptococcus salivarius* subsp. *thermophilus* *Bifidobacterium longum* subsp. *longum*	-Increase phenolic content	51.66–145.86 mg GAE g^−1^		[119]

Explanations: * not identified; TE—Trolox equivalent; RU—rutin equivalent; GAE—gallic acid equivalent; AE—ascorbic acid equivalent.

## Data Availability

Not applicable.
